# Significance of NotchScore and JAG1 in predicting prognosis and immune response of low-grade glioma

**DOI:** 10.3389/fimmu.2023.1247288

**Published:** 2023-11-13

**Authors:** Bo Shi, Fei Ge, Liangliang Cai, Yi Yang, Xiaohui Guo, Rui Wu, Zhehao Fan, Binjie Cao, Ning Wang, Yue Si, Xinyue Lin, Weibing Dong, Haibo Sun

**Affiliations:** ^1^ Institute of Translational Medicine, Medical College, Yangzhou University, Yangzhou, China; ^2^ School of Life Science, Liaoning Normal University, Dalian, Liaoning, China; ^3^ Jiangsu Key Laboratory of Experimental & Translational Non-Coding RNA Research, Yangzhou, Jiangsu, China; ^4^ Department of Gastroenterology, Haian Hospital of Traditional Chinese Medicine Affiliated to Nanjing University of Chinese Medicine, Nantong, Jiangsu, China

**Keywords:** notch, low-grade glioma, prognosis, tumor immune microenvironment, JAG1, PDL1

## Abstract

**Introduction:**

Low-grade glioma (LGG) is a prevalent malignant tumor in the intracranial region. Despite the advancements in treatment methods for this malignancy over the past decade, significant challenges still persist in the form of drug resistance and tumor recurrence. The Notch signaling pathway plays essential roles in many physiological processes as well as in cancer development. However, the significance of the pathway and family genes in LGG are poorly understood.

**Methods:**

We conducted gene expression profiling analysis using the TCGA dataset to investigate the gene set associated with the Notch signaling pathway. we have proposed a metric called "NotchScore" that quantifies the strength of the Notch signaling pathway and enables us to assess its significance in predicting prognosis and immune response in LGG. We downregulated JAG1 in low-grade gliomas to assess its influence on the proliferation and migration of these tumors. Ultimately, we determined the impact of the transcription factor VDR on the transcription of PDL1 through chip-seq data analysis.

**Results:**

Our findings indicate that tumors with a higher NotchScore, exhibit poorer prognosis, potentially due to their ability to evade the anti-tumor effects of immune cells by expressing immune checkpoints. Among the genes involved in the Notch signaling pathway, JAG1 has emerged as the most representative in terms of capturing the characteristics of both NotchScore and Notch pathways. The experimental results demonstrate that silencing JAG1 yielded a significant decrease in tumor cell proliferation in LGG cell lines. Our study revealed mechanisms by which tumors evade the immune system through the modulation of PDL1 transcription levels via the PI3K-Akt signaling pathway. Additionally, JAG1 potentially influences PDL1 in LGG by regulating the PI3K-Akt signaling pathway and the expression of the transcription factor VDR.

**Discussion:**

These findings contribute to our understanding of immune evasion by tumors in LGG. The insights gained from this research may have implications for the development of therapeutic interventions for LGG.

## Introduction

Glioma is a type of tumor that commonly develops within the central nervous system and has been extensively researched. The World Health Organization (WHO) has established a classification system for gliomas, which categorizes them into four grades according to their histopathological and molecular features (1, 2). Low-grade gliomas (LGG) are normally considered to have a low malignancy. However, LGG can still proliferate and progress in various ways, and the survival rate for patients with LGG is not ideal. According to a recent study, the median overall survival for grade II glioma patients with LGG is 78.1 months (2). Therefore, early diagnosis and treatment of LGG are crucial, and regular follow-up of LGG patients is necessary to detect any deterioration in their condition and to adjust treatment plans in a timely manner.

The Notch signaling pathway is a highly conserved pathway across evolution that contributes in regulating a number of cellular behaviors. These processes encompass cell proliferation, apoptosis, differentiation, tissue homeostasis maintenance, immune regulation, and disease progression (3, 4). Importantly, reports have discovered that blockade of the Notch pathway disrupts tumor blood vessel structure and promotes tumor metastasis (5). In osteosarcoma tissues and cells, there is a high expression of different molecules involved in the Notch signaling pathway (6). Specifically, the protein JAG1, belonging to the Notch family, exhibits significantly increased levels in highly metastatic osteosarcoma cells compared to low metastatic ones. Recent studies have demonstrated that suppressing JAG1 expression results in decreased proliferation, migration, and invasion capabilities of osteosarcoma cells (7).

As research on the tumor microenvironment (TME) continues, immunotherapy based on TME has been increasingly applied in clinical practice with promising results (8, 9). Recent studies have highlighted the close relationship between the biological characteristics of gliomas and their immune microenvironment (TME) (10). Glioma cells secrete diverse chemokines, cytokines, and growth factors that contribute in promoting immune cell infiltration into the tumor microenvironment. These cell types comprise most of the white blood cells including circulating progenitor cells, astrocytes, pericytes, endothelial cells, as well as various immune cells, including peripheral macrophages, microglia, effector T cells, and regulatory T cells (Treg cells) (11). Identifying these factors may aid in enhancing the immune modulation utilized by glioma cells, thereby serving as a basis for glioma immunotherapy (11, 12). In previous studies, mice with glioblastoma (GBM) were subjected to treatment using anti-PD-1 antibody or combinational therapies using anti-PD-1 and anti-CTLA-4 antibodies. Notably, both wild-type (WT) mice and CD73-/- mice receiving the combination therapy, which included anti-PD-L1, exhibited significantly improved survival rates compared to the control group (13). Hence, exploring the benefits of immune checkpoint inhibitors in LGG might be a viable approach.

Here, we studied the role of the Notch signaling pathway in low-grade gliomas (LGG). By analyzing the gene expression profiles of Notch signaling pathway-related genes in the TCGA dataset, we developed a metric called “NotchScore” to distinguish two distinct subtypes of LGG (CS1-CS2). Subsequently, we assessed the prognostic relevance of these two subtypes and investigated their disparities concerning the TME. Lastly, we conducted experimental validation to establish the critical role of the Notch family protein JAG1 in this process. These findings may provide a solid foundation for future research on targeted therapy for LGG.

## Results

### NotchScore clustering

**Figure 1:**
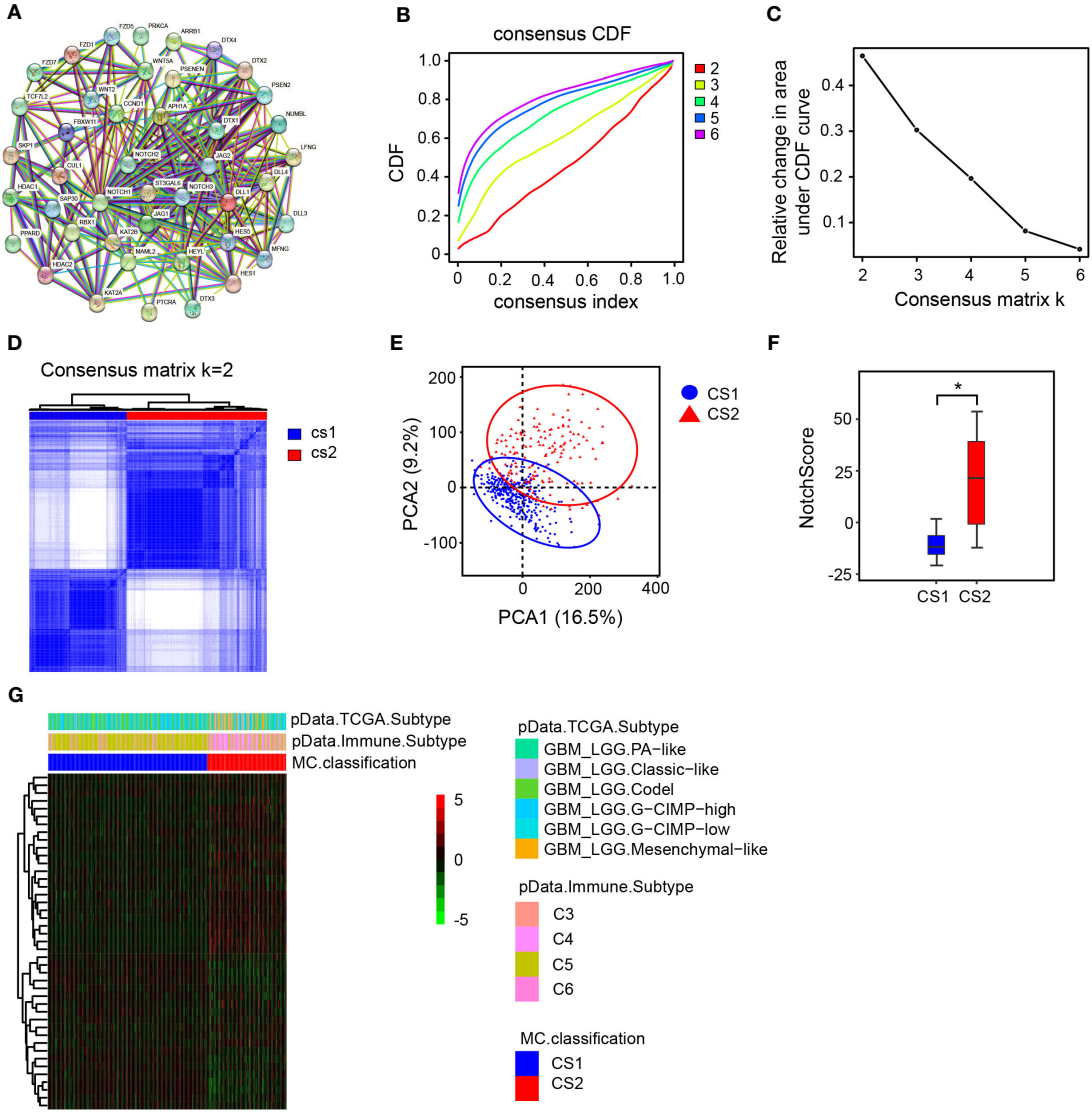
Cluster analysis and NotchScore. **(A)** Protein-protein interaction network diagram of Notch-related genes. **(B)** consensus matrix CDFs from K=2 to 6. The cumulative distribution functions are shown for varying values of k. CDF: cumulative distribution function. **(C)** Delta Area Plot, this graph illustrates the relative change in the area under the cumulative distribution function (CDF) curve when compared to k-1., **(D)** Heatmap of the matrix for k = 2. **(E)** Principal Component Analysis (PCA) based on mRNA expression patterns in LGG. PCA quantifies the differences between two groups of sample data by extracting two principal components, PC1 (Principal Component 1) and PC2 (Principal Component 2), which capture the largest and second-largest variations in the data, respectively. **(F)** Establishment of NotchScore to compare the differences between the two groups. **(G)** Heatmap illustrating the expression patterns of Notch-related genes in the two groups. PA-like: Based on the molecular similarity with pilocytic-astrocytomas. Classic-like: tumors belonging to the classical gene expression signature. Codel: consisting of IDH-mutant-codel LGGs. CIMP-high: IDH-mutant-non-codel glioma (LGG, GBM) manifesting relatively low genome wide DNA methylation. CIMP-low: IDH-mutant-non-codel glioma (LGG, GBM) with higher global levels of DNA methylation. Mesenchymal-like: enriched with mesenchymal subtype tumors.

In this study, we collected Notch signaling pathway-related genes from the HALLMARK database and previous studies. Utilizing the STRING database, we performed a protein-protein interaction (PPI) network analysis for identification of the crucial genes within the selected gene set and elucidate their interactions. We aimed to identify the core genes of the Notch signaling pathway within the selected gene set and gain a comprehensive understanding of their interactions. (**Figure 1A**). To explore the prognosis of LGG patients with different expression levels of Notch gene sets, unsupervised clustering analysis was performed on 483 LGG samples to determine the expression patterns of different groups of Notch gene sets. Cluster-consensus analysis and inter-group principal component analysis indicated that K=2 was the optimal cluster number, and thus the LGG samples were divided into two groups for further analysis (**Figures 1B–E**). The study established a NotchScore scoring method using the Genomic Grade Index to evaluate the expression of Notch-associated genes from each group of patients. These findings indicated that the CS2 group exhibited a higher NotchScore in comparison to the CS1 group (**Figure 1F**), and the heat map shows that samples in the CS2 group exhibit a diverse opposite patten of Notch gene expressions compared to CS1 group (**Figure 1G**).

### The NotchScore is related to LGG prognosis and specific signaling pathways

**Figure 2:**
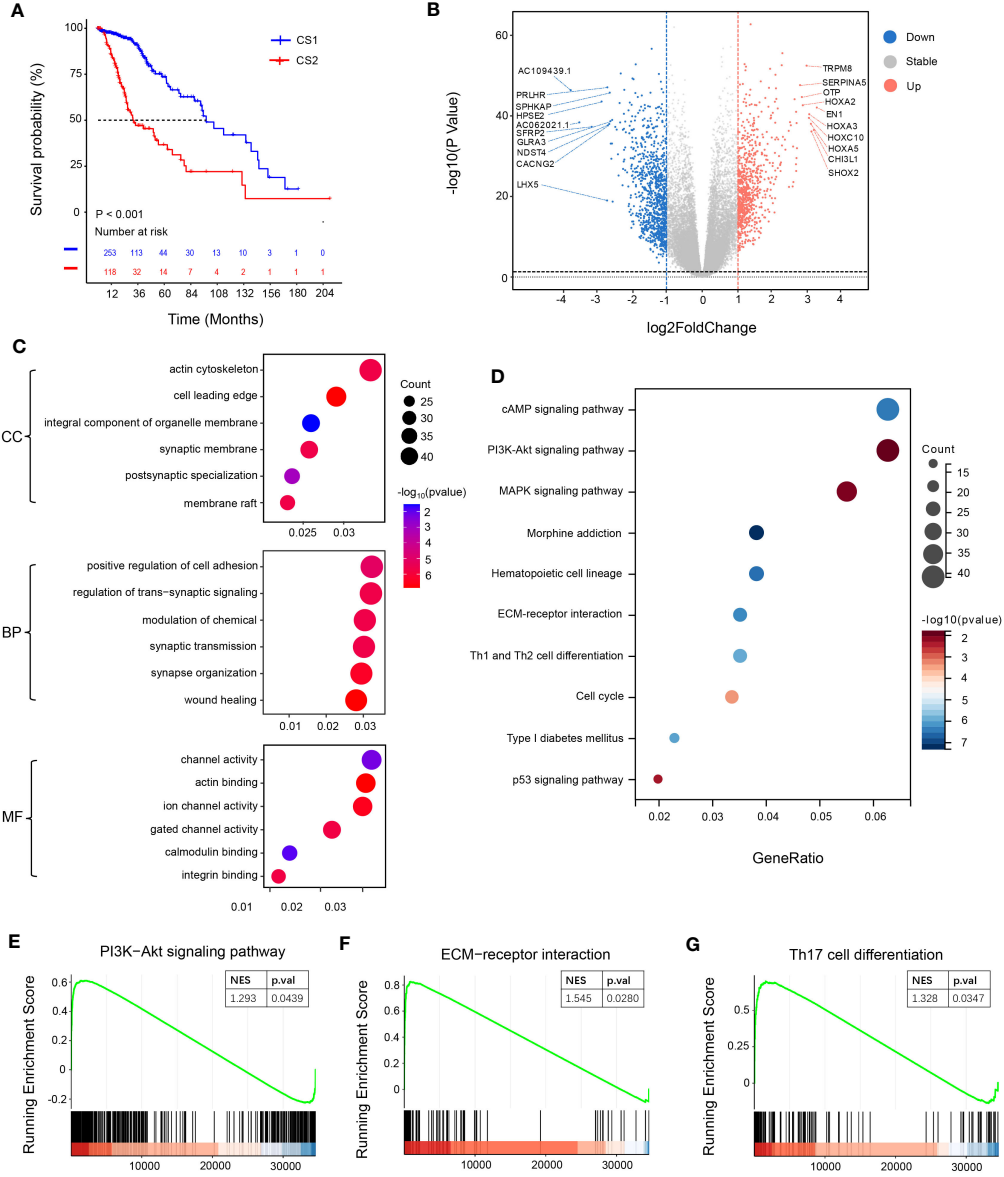
Survival analysis and functional specificity analysis of subgroups. **(A)** Kaplan-Meier curves of overall survival (OS) for the two groups after clustering analysis. **(B)** Volcano plot illustrating the distribution of differentially expressed genes. Genes with a P-value< 0.05 and |log2FoldChange| > 1 are considered differentially expressed, with 738 genes upregulated and 909 genes downregulated. **(C)** Gene Ontology (GO) enrichment pathway analysis. The x-axis of the bubble plot represents the proportion of differentially expressed genes in each pathway, while the y-axis represents the enriched pathways. “Count” indicates the number of differentially expressed genes enriched in each pathway. The color of the circles represents the -log10(P-value) of pathway enrichment. **(D)** Kyoto Encyclopedia of Genes and Genomes (KEGG) enrichment pathway analysis. **(E–G)** Pathway analysis using the gene set enrichment analysis (GSEA) method (CSEA).

To examine the correlation between NotchScore and prognosis of LGG patients, survival analysis was performed using TCGA dataset. The results showed that patients in the CS2 group, characterized by high NotchScore, had a poorer prognosis compared to those in the CS1 group, characterized by low NotchScore (**Figure 2A**).To further explore the association between the mRNA levels of Notch-associated genes and prognosis, we conducted differential gene analysis. This analysis involved applying specific criteria, including a fold change (FC) threshold of >2 or<-2, |log_2_FC|>1, and significant p-values (p.val)< 0.05, -log_10_ (p.val) >-log_10_ 0.05. As a result, we identified 738 upregulated differentially expressed genes (DEGs) and 909 downregulated DEGs (**Figure 2B**).We performed enrichment analysis on the DEGs to identify relevant biological functions and signaling pathways. The gene ontology (GO) analysis revealed enrichment in channel activity, positive regulation of cell adhesion, and actin cytoskeleton. Additionally, the Kyoto Encyclopedia of Genes and Genomes (KEGG) analysis identified several important signaling pathways, including the p53, cAMP, MAPK, cell cycle, and PI3K-Akt signaling pathways (**Figures 2C, D**). Moreover, we conducted gene set enrichment analysis (GSEA) and observed that the CS2 group exhibited upregulation of the ECM-receptor interaction, PI3K-Akt signaling pathway, and Th17 cell differentiation (**Figures 2E–G**).

### NotchScore and Notch-related genes are correlated with TME

**Figure 3:**
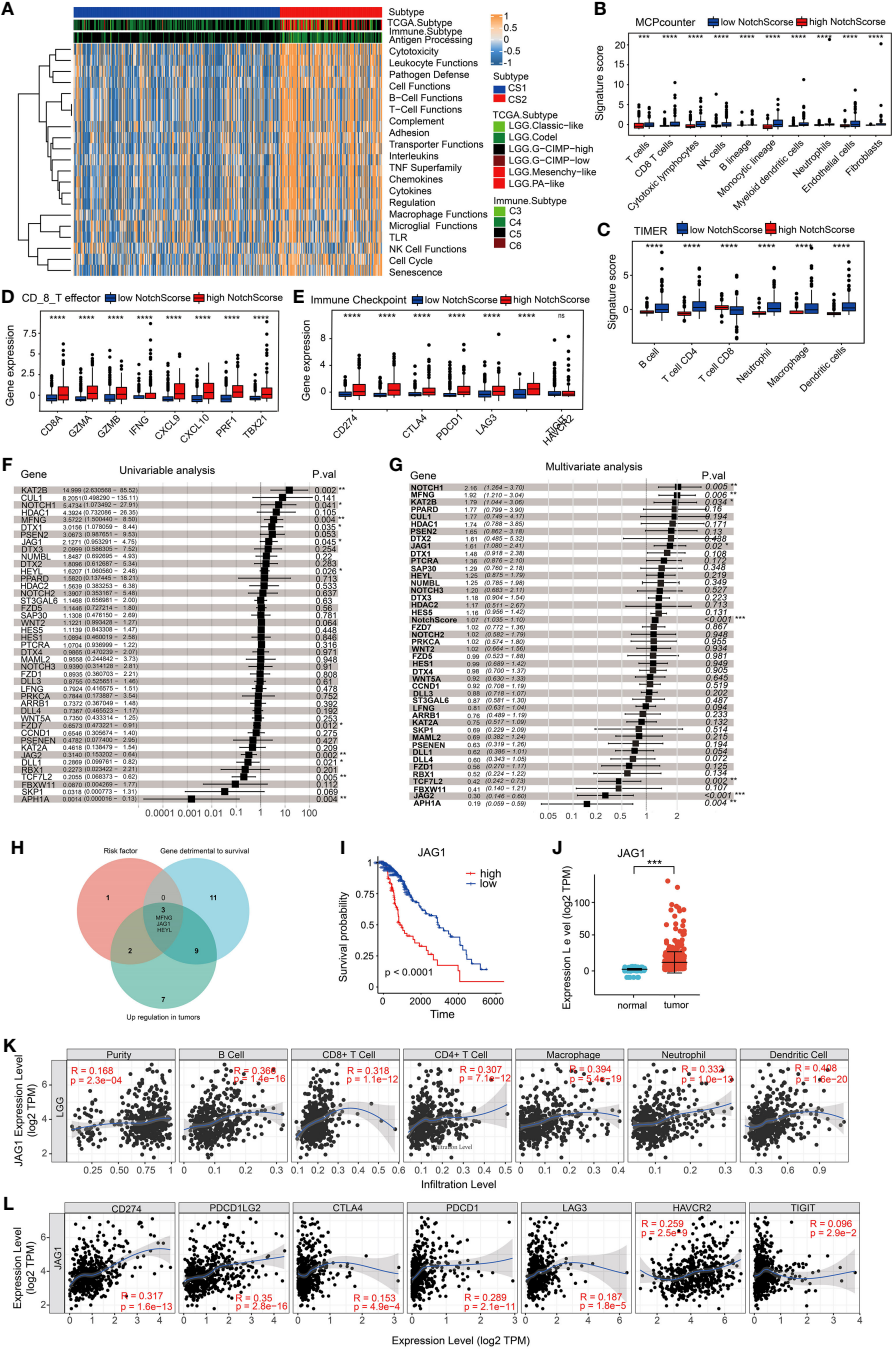
Immune characteristics of two subclasses in the metadata set. **(A)** Evaluation of pathway/function activity changes using Gene Set Variation Analysis (GSVA). **(B, C)** Analysis of differences in immune cell abundance between different groups using two methods: MCPcounter and TIMER. **(D, E)** Expression levels of CD8_T effector factors and immune checkpoints. The x-axis represents different genes, while the y-axis represents gene expression levels. **(F)** Univariable cox analysis. **(G)** Univariable cox analysis. **(H)** Venn diagram illustrating three genes that meet the criteria for risk factors, with higher gene expression levels in tumors. High expression of these genes in patient samples is associated with poor prognosis. **(I)** Kaplan-Meier curve demonstrating the expression of JAG1 and overall survival (OS) in LGG patients. **(J)** Differential expression of JAG1 between normal brain tissue and LGG. **(K, L)** Correlation between JAG1 expression and immune infiltration levels, as well as the expression of immune checkpoints. *:P<0.05, **:P<0.01, ***P<0.001.

Recent studies have demonstrated that T cell antigen receptor (TCR), CD28, and interleukin-2 receptor activate PI3K through the phosphorylation and deactivation of PI3K inhibitory molecules. PI3K converts PIP2 into PIP3, which in turn recruits downstream signaling molecules such as PDK1 and Akt to the membrane and activates them (14). mTORC2 further activates Akt and promotes metabolic expansion and T cell effector function (14, 15). The enrichment pathway analysis showed significant enrichments of the PI3K-Akt and Th1 and Th2 cell differentiation signaling pathways, indicating notable differences in immune function between the two groups. Using GSVA gene set variation analysis, the study found that compared to the CS1 group, the CS2 group had an upregulation trend in antigen presentation, white blood cell function, lymphocyte functions (B cell, T cell, and NK cell), and cell cycle (**Figure 3A**). These findings imply potential variations in the tumor immune microenvironment between the CS1 and CS2 groups. Therefore, the study analyzed related immune factors such as immune infiltration and immune checkpoints. MCPcounter and TIMER were utilized to assess the immunoheterogeneity between these subtypes and provide a comprehensive overview of distinct immune cell infiltrations. Specifically, compared to samples with low NotchScore, samples with high NotchScore showed low levels of immune cell infiltration, including T cells, cytotoxic lymphocytes, macrophages, NK cells, and B cells (**Figures 3B, C**). Furthermore, immune checkpoint and CD8 T cell effectors were significantly upregulated in the group with higher NotchScore (**Figures 3D, E**). The TIDE Score Calculation, utilized for evaluating the potential clinical effectiveness of immunotherapy in various risk groups, mirrors the potential tumor immune evasion capability (**Figure S1-B**). The analysis results suggest an elevated probability of immune evasion in patients with a high NotchScore. Univariable analysis and multivariate analysis of the Notch gene set showed that genes were identified as risk factors for the CS2 group (**Figures 3F, G**). The NotchScore value exhibits statistical significance in its impact on survival time(HR=1.07, 95%CI 1.035-1.10, P<0.001) (**Figure 3G**). We then set the criteria for gene selection, focusing on genes that were not only risk factors for the CS2 group but also exhibited higher expression levels in tumors and their high expression in patient was associated with poor prognosis. Venn diagram analysis revealed that three genes, namely JAG1, MFNG, and HEYL, met these criteria (**Figure 3H**). The data analysis revealed a significant correlation between high JAG1 expression and poor prognosis in patients. Additionally, the expression of JAG1 in tumor tissues is elevated compared to normal brain tissues (**Figures 3I, J**). Specifically, JAG1 expression showed a positive correlation with the immune cell infiltrations of various cell types, including B cells, T cells, neutrophils, macrophages and dendritic cells. Additionally, There is a positive correlation between mRNA levels of JAG1 and various immune checkpoint markers, including CD274, PDCD1, PDCD1-LG2, and HAVCR2 (**Figures 3K, L**). These findings show that JAG1 may be a new target for detecting sensitivity to tumor and immune therapy, as well as for tumor treatment.

### JAG1 silencing reduces Notch signaling transduction

**Figure 4:**
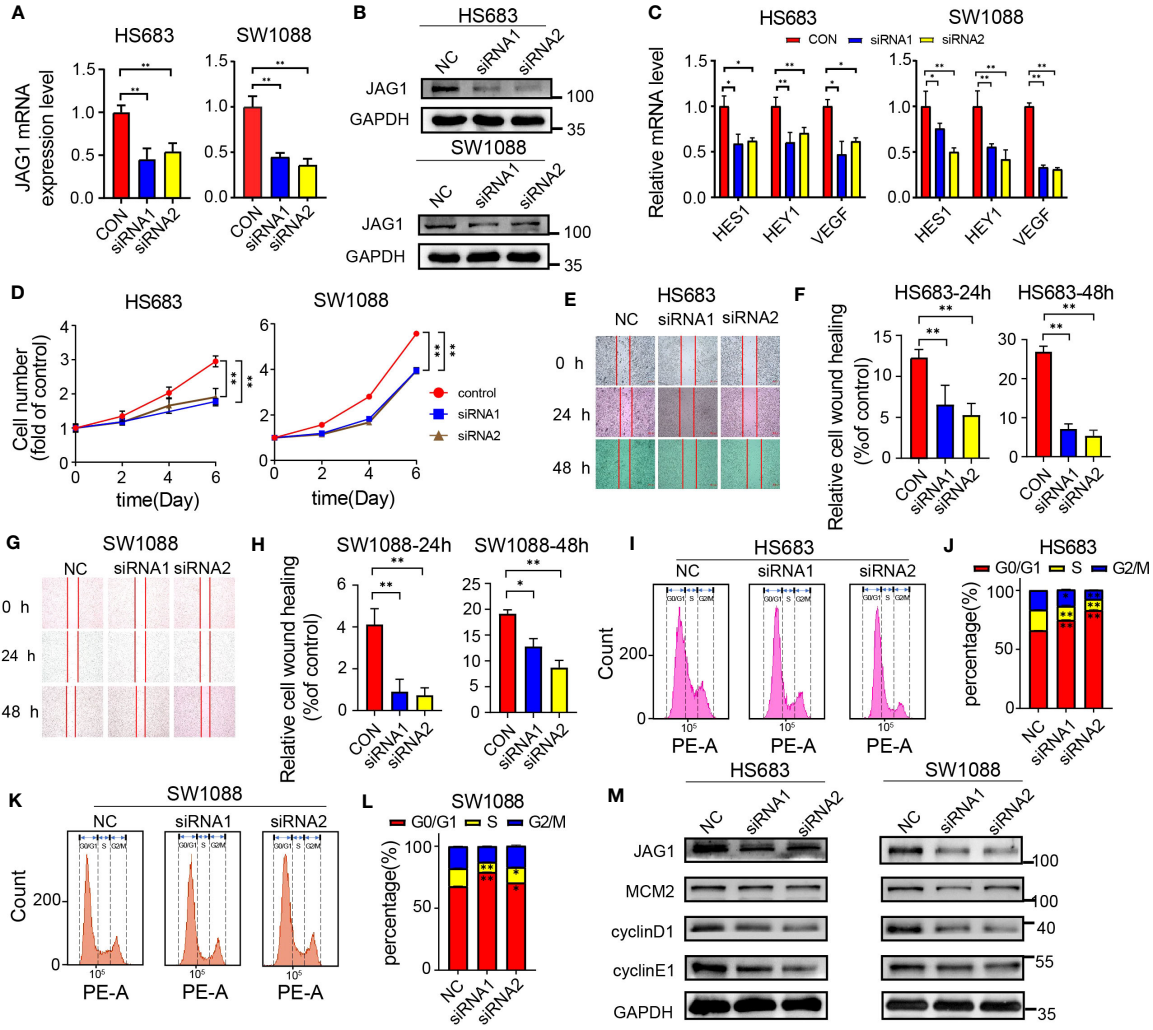
JAG1 regulates the proliferation and migration of LGG cells. **(A)** qPCR results confirm the effective downregulation of JAG1 in HS683 and SW1088 cells. **(B)** Western blot results validate the successful silencing of JAG1 in HS683 and SW1088 cells. **(C)** qPCR analysis reveals the expression levels of downstream factors (HES1, hey1, VEGF) in the Notch signaling pathway at the RNA level. **(D)** Impact of JAG1 silencing on the proliferation of HS683 and SW1088 cells. **(E, G)** Effect of JAG1 silencing on the migration capability of SW1088 and HS683 cells. **(F, H)** Bar graphs depicting cell migration rates at 24 hours and 48 hours. **(I, K)** Flow cytometry analysis investigating the influence of JAG1 silencing on the cell cycle of HS683 and SW1088 cells. **(J, L)** Bar graphs illustrating the distribution of cells in the G0/G1, S, and G2/M phases. **(M)** Western blot analysis of key cell cycle regulators. *:P<0.05, **:P<0.01, ***P<0.001.

To evaluate the effects of JAG1 silencing on the cell proliferation and migration capabilities in low-grade glioma (LGG) cells, we employed small interfering RNA (siRNA) specifically designed for JAG1 and transfected it into SW1088 and HS683 cell lines. Subsequently, we examined the expression of JAG1 after siRNA interference and confirmed that JAG1 expression was effectively suppressed both at the mRNA and protein levels (**Figures 4A, B**). The downstream factors of Notch signaling pathway including Hes1, Hey1, and VEGF of the were also examined, and qPCR results showed that their transcription levels were decreased after JAG1 silencing, indicating that Notch signal transduction was inhibited (**Figure 4C**). MTT assays were further conducted in order to evaluate the impact of JAG1 silencing on cell proliferation. The results demonstrated a notable reduction in the proliferation capacity of HS683 and SW1088 cells compared to the control group. (**Figure 4D**). Scratch assays were thereafter conducted to test JAG1 silencing on the migration ability and results revealed a decrease in the migration ability of HS683 and SW1088 cells following JAG1 silencing. (**Figures 4E–H**). Furthermore, changes in the cell cycle of HS683 and SW1088 cells after JAG1 silencing were examined. The results indicated a rise in the population of cells in the G1 phase, together with a reduction in cells in the S and G2 phases. These findings suggest a potential cell cycle arrest effect. (**Figures 4I–L**). Additionally, immunoblot results showed that core cell cycle regulating factors such as MCM2, cyclin D1 and cyclin E1 were downregulated at the protein level after JAG1 silencing (**Figure 4M**).

### JAG1 expression regulates immune checkpoint-related genes

**Figure 5:**
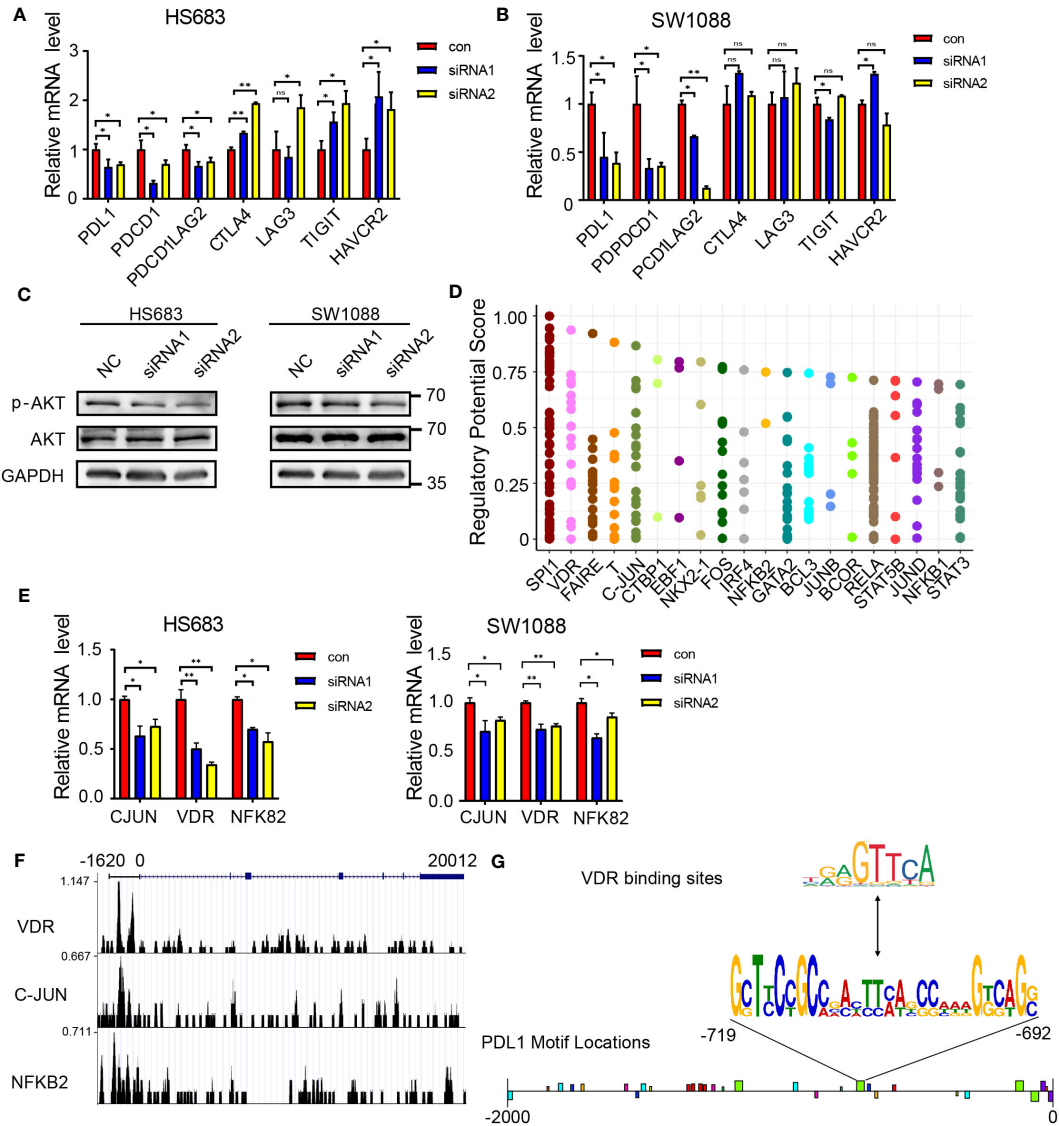
JAG1 regulates PDL1 through the PI3K-AKT pathway and VDR. **(A, B)** qPCR analysis reveals the changes in mRNA expression of immune checkpoint genes following JAG1 silencing. **(C)** Western blot analysis demonstrates the protein level alterations of key factors (p-AKT and AKT) in the PI3K-AKT signaling pathway upon JAG1 silencing. **(D)** Prediction of transcription factors regulating PDL1 using cistrome DB. **(E)** qPCR analysis shows the changes in mRNA expression of transcription factors after JAG1 silencing. **(F)** Chip-seq analysis investigates the binding sites of transcription factors with PDL1. **(G)** Motif analysis predicts the binding sites of PDL1 with VDR. *:P<0.05, **:P<0.01, ***P<0.001.

To examine the association between JAG1 and immune checkpoints, this research assessed the mRNA levels of immune checkpoints. The findings revealed a noteworthy reduction in the expression of PDL1 and PDCD1 upon JAG1 knockdown. However, the expression levels of other immune checkpoints remained unaltered. (**Figures 5A, B**). Previous research has indicated that the AKT-mTOR pathway promotes immune evasion in lung adenocarcinoma by driving PD-L1 expression (16). Furthermore, the Notch signaling pathway interacts with the PI3K-AKT pathway to some extent. In this study, we indeed found a strong significance of the PI3K-AKT signaling pathway in differential gene enrichment pathways and P-AKT levels were decreased after JAG1 knockdown (**Figure 5C**). To investigate the transcriptional regulatory relationship between JAG1 and PDL1, this study predicted the transcription factors of PDL1 through cistrome DB (**Figure 5D**), and then selected the top 10 ranked transcription factors for mRNA-level detection. The qPCR results showed that among the 10 transcription factors, C-JUN, NFKB2, and VDR were downregulated at the mRNA level in HS683 and SW1088 cells when JAG1 was silenced (**Figure 5E**). Additionally, we reanalyzed previous reported CHIP-seq data of C-JUN (GSM1208639), NFKB2 (GSM1208776), and VDR (GSM791404), and discovered that VDR may exhibit a higher binding peak by binding to the promoter region of PDL1 (**Figures 5F, G**). Simultaneously, we observed distinct binding peaks of VDR in the PDL1 promoter region across various tissues (**Figures S1-C**). Since VDR has been shown to regulate PDL1 transcription (17, 18), our preliminary results indicate that JAG1 may activate PDL1 transcription through up-regulation of VDR.

## Discussion

The Notch signaling pathway is a conserved signaling pathway that depends on direct cell-to-cell contact between cells of the same type or different types for signal transmission (7). The Notch receptor and ligand can be expressed on the same cell or on different cells (3). Due to its robust intercellular communication, the Notch signaling pathway plays an important role in regulating and controlling cell fate. The Notch pathway has been associated with various types of cancer including brain cancer, breast cancer, and lung cancer, among others (3). This provides a solid theoretical basis for further investigating the Notch signaling pathway in low-grade glioma (LGG). Our analysis revealed that high expression levels of Notch-related genes are linked to poor survival and reduced immune infiltration. These findings suggest that the Notch signaling pathway is vital for immune cell differentiation, tumor microenvironment formation, and tumor development. Analysis using COX regression and a comparison of Notch gene expression between normal tissues and tumors revealed that JAG1 expression is elevated in tumors and is positively associated with a poorer prognosis. Moreover, JAG1 exhibits a strong correlation with the immune response. These results suggest the importance of JAG1 in LGG.

In brain and ovarian cancer, JAG1 is strongly expressed in tumor-associated blood vessels, suggesting its involvement in angiogenesis (19). Previous studies have demonstrated that increased JAG1 expression activates the Notch signaling pathway, promoting the proliferation of invasive cancer cells in adrenocortical carcinoma (20). Interestingly, a previous immunohistochemical assay showed an increase in JAG1 expression with higher WHO grades of gliomas (levels from II to IV) (21), which indicates JAG1 protein expression maybe associated with progression of the disease. In the context of LGG, experimental results indicate that silencing JAG1 reduces the mRNA expression level of VEGF, a key regulator of angiogenesis, and weakens the migration ability of HS683 and SW1088 cells (3). Cell cycle analysis revealed that JAG1 knockdown led to an increased proportion of cells in the G0/G1 phase. Moreover, the protein levels of cell cycle regulatory proteins, such as cyclin D1 and cyclin E1, were reduced. These proteins has been recognized to regulate the transition of G1 to S phase of the cell cycle. These findings suggest that JAG1 potentially influences the proliferation and migration of LGG cells by modulating downstream activity of the Notch pathway and cell cycle regulatory proteins, thereby promoting tumor progression.

In addition, the researchers found that soluble monomeric JAG1 signaling significantly reduced the immunosuppressive function of Treg cells and increased anti-tumor immunity, further highlighting the crucial role of Notch signaling and its related ligands in immunity (22). The study also showed that silencing JAG1 significantly down-regulated the mRNA levels of PDL1 and PDCD1 in LGG cells. It has been reported that the activation of the PI3K-AKT, Wnt, and EGFR pathways in glioma samples can all promote the upregulation of PD-L1 expression levels (14, 15). Consistent with these findings, we confirmed the upregulation of PD-L1 in LGG cells at the protein level. To investigate the regulatory mechanism of JAG1 on PDL1, we employed cistrome DB to screen for potential transcription factors that could bind to the PDL1 gene. After silencing JAG1, the mRNA levels of JUN, NFKB2, and VDR exhibited a significant decrease. Using the transcription factor prediction website JASPAR, we predicted the binding motifs of VDR with JUN, NFKB2, and VDR, and their binding with the 5’ promoter region of PDL1. The results showed multiple predicted binding sites of VDR in the PDL1 promoter region. Overall, our findings suggest that JAG1 may affect the transcription of PDL1 by regulating the expression of VDR, leading to tumor immune escape, promoting tumor cell survival, and shortening patient survival.

Our study has several limitations. Tumors can exhibit diverse characteristics in their external and internal microenvironments. Treating the tumor as a whole may not effectively differentiate the Notch expression levels and immune status across different tumor locations. To address this, future studies can explore the application of single-cell RNA sequencing in combination with spatial transcriptomics analysis. Moreover, it’s worth noting that the data and tissues utilized in this study were obtained from public databases, which may not encompass all the changes occurring in all relevant regions of LGG cases. Therefore, it is crucial to analyze multiple datasets from various sources and validate the results. This should be the focus of future research.

In summary, our LGG classification based on the Notch gene set provides a clear description of the heterogeneity of the immune microenvironment in different LGGs. Our study also demonstrates that JAG1 affects the proliferation of LGG cell lines and PDL1 expression, thus influencing tumor development. This provides valuable insights for the development of therapeutic drugs targeting LGG.

## Materials and methods

### Data collection

In this study, the clinical and RNAseq data of 483 LGG patients was obtained from the TCGA database. As normal brain tissue samples were not available in the TCGA LGG dataset, we downloaded 207 RNA-seq datasets of normal brain tissues as controls from the GTEX database. Therefore, a total of 483 LGG and 207 normal samples were included for the analysis. To mitigate the influence of batch effects, this study employed ComBat from SVA to process the data. The ChIP-seq data is derived from cistrome DB (http://cistrome.org/db/).

### Protein interaction network

This study analyzed the Notch pathway in low-grade glioma (LGG) by selecting 43 relevant genes from the HALLMARK database. Using the R package “Consensus-ClusterPlus” with parameters reps=1000 and pItem=0.8, the 483 LGG patients were clustered based on the mRNA levels of these genes. Principal component analysis (PCA) was conducted to visualize classification differences among the identified clusters. PCA were compared using the R packages “FactoMineR”. Survival curves were compared using the R packages “survival” and “jskm”. The STRING database was utilized to construct and visualize the interaction interface of the Notch-related gene set, while GeneMania was used to visualize protein connections.

### NotchScore

In this study, the NotchScore for each patient was calculated using unsupervised clustering based on Notch pathway-related gene expression. Common differentially expressed genes (DEGs) were captured between the clustered groups. The Notch Score was defined using the Genomic Grade Index (GGI). It involved multiplying the weights of DEGs with significant differential expression in the Notch pathway-related gene cluster (PC1/PC2) by the GGI (*i*) and summing them to obtain the patient’s Notch Score. The NotchScore was then used to assess Notch pathway activity and its relationship with LGG prognosis.


NotchScore=∑(PC1i+PC2i)

### Functional enrichment analysis and TME analysis

This study utilized various analytical techniques to investigate cancer hallmark pathways in LGG patients. The techniques included GSVA and GSEA for pathway analysis, GO and KEGG enrichment analyses for gene annotation, TME analysis using the ESTIMATE package, quantification of immune cell infiltration with ssGSEA, and evaluation of immune checkpoints. The functional enrichment analysis and TME analysis were compared using the R packages “GSEABase”, “limma” and “clusterProfiler”. The TIDE Score is calculated through the TIDE online calculation website (http://tide.dfci.harvard.edu/).

### Cell culture and treatment

All cell lines were cultured under conditions recommended by product instructions. HS683 (Yaji Biotechnology, Shanghai, China) and SW1088 cell (Tongpai Biotechnology, Shanghai, China) were both cultured in DMEM medium with 10% FBS. The se-quence of siRNA can be found in **Supplementary Table S1** (Geneseeq Technology, Su-zhou, China). Each well of the 96-well plate was seeded with 5000 cells and treated with MTT mixed solution on days 0, 2, 4 and 6, followed by 4-hour incubation. After removing the supernatant, 120μl of DMSO was supplied to dissolve the sediment, and the OD value was measured (490 nm wavelength) to detect cell proliferation. When the number of cells reaches 2*106 in the 6-well plate, the cells should be digested, fixed with 70% ethanol, and then incubated with PI dye for detecting the cell cycle using a flow cy-tometer.

### Western blotting

Standard Western blotting assay was performed using rabbit polyclonal anti-JAG1, anti-P27, anti-MCM2, anti-cyclin D1, anti-cyclin E1, and mouse monoclonal anti-GAPDH purchased (Sanying Biotechnology, Wuhan, China). Rabbit polyclonal anti-pAKT and anti-AKT were obtained (Cell Signaling Technology, Massachusetts, USA). The protein levels were normalized, and quantification analysis was executed using ImageJ. The bar graph shows fold changes.

### RNA extraction, reverse transcription, and qPCR

In this study, TRNzol was used to extract total RNA from HS683 and SW1088 cells (Tiangen Biotech, Beijing, China). The extracted RNA was reverse transcribed into cDNA using HiScript III RT SuperMix with gDNA wiper (Vazyme Biotech, Nanjing, China). RT-QPCR was performed using the ABI 7900 RT-PCR system. The relative mRNA levels of the genes were calculated using the 2^-ΔΔCt^ method, with GAPDH serving as the internal reference. The primers used in the study can be found in **Supplementary Table S2**.

### Statistical analysis

The statistical analyses were conducted using RStudio. Independent sample t-tests were utilized to analyze normally distributed continuous variables. Apply the Kaplan-Meier method to analyze the survival of the samples. Statistical significance was considered as a p-value< 0.05. In the text, p-values are indicated using asterisks “*”. *:P<0.05, **:P<0.01, ***P<0.001. QPCR, cell proliferation and cell cycle experiment were performed three independent times.

## Data availability statement

The original contributions presented in the study are included in the article/**Supplementary Materials**, further inquiries can be directed to the corresponding author/s.

## Ethics statement

Ethical approval was not required for the studies on humans and animals in accordance with the local legislation and institutional requirements because only commercially available established cell lines were used.

## Author contributions

HS and WD designed this study. BS, FG, LC, YY, XG, RW, ZF, BC, NW, YS, and XL performed the experiments and acquired the data. BS performed the bioinformatic analysis and analyzed experimental data. BS drafted the manuscript. All authors contributed to the article and HS edited and approved the submitted version.
