# Supplementary material for: Significance of NotchScore and JAG1 in predicting prognosis and immune response of low-grade glioma
Source: Front Immunol. 2023 Nov 13;14:1247288. doi: 10.3389/fimmu.2023.1247288 (PMC10679421; doi:10.3389/fimmu.2023.1247288)
Supplement: Supplementary file 1 [file DataSheet_1.pdf]

Table S1. JAG1 siRNAs synthesized in the study

Gene	sense（5’-3’）	antisense（5’-3’）
Negative control(human)	UUCUCCGAACGUGUCACGUTT	ACGUACACGUUCGGAGAATT
JAG1-1(human)	CAUCGAUUAUUGUGAGCCUTT	AGGCUCACAAUAAUCGAUGTT
JAG1-2(human)	CCUGUAACAUAGCCCGAAATT	UUUCGGGCUAUGUUACAGGTT
