# Supplementary material for: Significance of NotchScore and JAG1 in predicting prognosis and immune response of low-grade glioma
Source: Front Immunol. 2023 Nov 13;14:1247288. doi: 10.3389/fimmu.2023.1247288 (PMC10679421; doi:10.3389/fimmu.2023.1247288)
Supplement: Supplementary file 2 [file DataSheet_2.pdf]

Table S2. RT-qPCR primers used in the study

Gene	Primer（5'-3'）	Reverse primer（5'-3'）
JAG1	TGCCAAGTGCCAGGAAGT	GCCCCATCTGGTATCACACT
HES1	GGACATTCTGGAAATGACAGTGA	AGCACACTTGGGTCTGTGCTC
hey1	CCGAGATCCTGCAGATGACC	CCCGAAATCCAAACTCCGA
VEGF	TCTACCTCCACCATGCCAAGT	GATGATTCTGCCCTCCT
PD-L1	TGGCATTTGCTGAACGCATTT	TGCAGCCAGGTCTAATTGTTTT
CTLA4	TCTTCATCCCTGTCTTCT	ATACTCACACACAAAGCTG
TIGIT	TGCCAGGTTCCAGATTCCATTGC	GCGACCACCACGATGACTGC
HAVCR2	AATACAGAGCGGAGGTCGGTCAG	GTTGCCACATTCAAACACAGGACAG
LAG3	CACCTCCTGCTGTTTCTCATCCTTG	TGTCCCTGGCTCACCTGTCTTC
PDCD1	GTGCCTGTGTTCTCTGTGGACTATG	TGAGGTGCCCATTCCGCTAGG
PDCD1LG2	GGACTCACCTCTGGAGCCTATGG	TTTCAGTCTGGCAGCAAGAAGGATC
c-Jun	GCTGGAGCGCCTGATAATCCAGTC	CCTGCTCATCTGTCACGTTCTTG
SPI1	ATGGAAGGGTTTCCCCTCGT	CTGGAGCTCCGTGAAGTTGT
VDR	AGACCTCACAGAAGAGCACC	CCATTGCCTCCATCCCTGAA
TBXT	CCCGTCTCCTTCAGCAAAGTC	TGGATTCGAGGCTCATACTTATGC
CTBP1	TACAGCGAGCAGGCATCC	TGGTCCTTGTTGACACAGTTC
EBF1	GGTTTCCCGCATTCTTTAGG	GTGGCAACCGAAATGAGACT
FOS	CCGGGGATAGCCTCTCTTAC	GTGGGAATGAAGTTGGCACT
Irf4	AGATTCCAGGTGACTCTGTG	CTGCCCTGTCAGAGTATTTC
NFKB2	GGCAGACCAGTGTCATTGAGCA	CAGCAGAAAGCTCACCACACTC
GATA2	CAAGGCTCGTTCCTGTTCAG	TGCCCATTCATCTTGTGGTAG
