# Supplementary material for: Significance of NotchScore and JAG1 in predicting prognosis and immune response of low-grade glioma
Source: Front Immunol. 2023 Nov 13;14:1247288. doi: 10.3389/fimmu.2023.1247288 (PMC10679421; doi:10.3389/fimmu.2023.1247288)
Supplement: Supplementary file 3 [file DataSheet_3.pdf]

Table S3-1. Translation of Abbreviations and Full Names

abbreviation	full name
JAG1	jagged canonical Notch ligand 1
MCM2	minichromosome maintenance complex component 2
AKT	AKT serine/threonine kinase 1
PDL1	CD274 molecule
VDR	vitamin D receptor
C-JUN	Jun proto-oncogene
NFKB2	nuclear factor kappa B subunit 2
MFNG	MFNG O-fucosylpeptide 3-beta-N-acetylglucosaminyltransferase
HEYL	hes related family bHLH transcription factor with YRPW motif like
PDCD1	programmed cell death 1
PDCD1-LG2	programmed cell death 1 ligand 2
HAVCR2	hepatitis A virus cellular receptor 2
MAPK	mitogen-activated protein kinase
PI3K	Phosphoinositide 3-kinase
PIP2	Phosphatidylinositol (4,5)bisphosphate
PIP3	interaction protein for cytohesin exchange factors 1
mTOR	mammalian target of rapamycin

Table S3-2. catalog numbers of antibodies

antibodies	catalog numbers
anti-JAG1	Cat No. 66890-1-Ig
anti-P27	Cat No. 25614-1-AP
anti-MCM2	Cat No. 10513-1-AP
anti-cyclin D1	Cat No. 26939-1-AP
anti-cyclin E1	Cat No. 11554-1-AP
anti-GAPDH	Cat No. 10494-1-AP
anti-pAKT	To Purchase # 4060S
anti-AKT	To Purchase # 4685S
