# Supplementary material for: Significance of NotchScore and JAG1 in predicting prognosis and immune response of low-grade glioma
Source: Front Immunol. 2023 Nov 13;14:1247288. doi: 10.3389/fimmu.2023.1247288 (PMC10679421; doi:10.3389/fimmu.2023.1247288)
Supplement: Supplementary file 4 [file DataSheet_4.pdf]

Supplemental Figure-1

A: ROC（Receiver Operating Characteristic）, NotchScore holds significant diagnostic value for cancer patient progression.

B: The TIDE Score Calculation, utilized for evaluating the potential clinical effectiveness of immunotherapy in various risk groups, mirrors the potential tumor immune evasion capability.

C: Chip-seq analysis investigates the interaction of VDR with PDL1 transcriptional regulatory sites in various tissues.
