# Supplementary material for: Significance of NotchScore and JAG1 in predicting prognosis and immune response of low-grade glioma
Source: Front Immunol. 2023 Nov 13;14:1247288. doi: 10.3389/fimmu.2023.1247288 (PMC10679421; doi:10.3389/fimmu.2023.1247288)
Supplement: Supplementary file 6 [file Table_1.docx]

**Raw Data link**

https://www.jianguoyun.com/p/DSO4JSkQv47kCxjP7JAFIAA
